# Effects of Introducing Xpert MTB/RIF on Diagnosis and Treatment of Drug-Resistant Tuberculosis Patients in Indonesia: A Pre-Post Intervention Study

**DOI:** 10.1371/journal.pone.0123536

**Published:** 2015-06-15

**Authors:** Sanne C. van Kampen, Nugroho H. Susanto, Sumanto Simon, Shinta D. Astiti, Roni Chandra, Erlina Burhan, Muhammad N. Farid, Kendra Chittenden, Dyah E. Mustikawati, Bachti Alisjahbana

**Affiliations:** 1 Access to Laboratory Services Team, KNCV Tuberculosis Foundation, The Hague, the Netherlands; 2 Medical Faculty, Universitas Padjadjaran, Bandung, Indonesia; 3 Tuberculosis Operational Research Group, Ministry of Health, Jakarta, Indonesia; 4 Medical Faculty, Universitas Atmadjaja, Jakarta, Indonesia; 5 Laboratory Team TB CARE I, KNCV Tuberculosis Foundation, Jakarta, Indonesia; 6 Department of Lung and Respiratory Health, Persahabatan Hospital, Jakarta, Indonesia; 7 Sub-Directorate Statistics and Design, Central Bureau of Statistics, Jakarta, Indonesia; 8 Health Division, United States Agency for International Development, Jakarta, Indonesia; 9 National Tuberculosis Control Program, Ministry of Health, Jakarta, Indonesia; McGill University, CANADA

## Abstract

**Background:**

In March 2012, the Xpert MTB/RIF assay (Xpert) was introduced in three provincial public hospitals in Indonesia as a novel diagnostic to detect tuberculosis and rifampicin resistance among high risk individuals.

**Objective:**

This study assessed the effects of using Xpert in place of conventional solid and liquid culture and drug-susceptibility testing on case detection rates, treatment initiation rates, and health system delays among drug-resistant tuberculosis (TB) patients.

**Methods:**

Cohort data on registration, test results and treatment initiation were collected from routine presumptive patient registers one year before and one year after Xpert was introduced. Proportions of case detection and treatment initiation were compared using the Pearson Chi square test and median time delays using the Mood’s Median test.

**Results:**

A total of 975 individuals at risk of drug-resistant TB were registered in the pre-intervention year and 1,442 in the post-intervention year. After Xpert introduction, TB positivity rate increased by 15%, while rifampicin resistance rate reduced by 23% among TB positive cases and by 9% among all tested. Second-line TB treatment initiation rate among rifampicin resistant patients increased by 19%. Time from client registration to diagnosis was reduced by 74 days to a median of a single day (IQR 0–4) and time from diagnosis to treatment start was reduced by 27 days to a median of 15 days (IQR 7–51). All findings were significant with p<0.001.

**Conclusion:**

Compared to solid and liquid culture and drug-susceptibility testing, Xpert detected more TB and less rifampicin resistance, increased second-line treatment initiation rates and shortened time to diagnosis and treatment. This test holds promise to improve rapid case finding and management of drug-resistant TB patients in Indonesia.

## Introduction

In December 2010, the World Health Organization (WHO) recommended the use of Xpert MTB/RIF (Xpert) as a new automated molecular test to rapidly and simultaneously detect TB and rifampicin resistant (RR-)TB, which can be a good proxy for multidrug-resistant (MDR-)TB [1–3]. Xpert is recommended to be used as the initial test for individuals at risk of MDR-TB, because it has similar accuracy to that of conventional culture and drug-susceptibility testing (DST) for rifampicin (RIF) and provides results within two hours instead of weeks (liquid media) or months (solid media) [4, 5]. While large scale demonstration studies have shown that Xpert introduction is feasible in high-burden countries under project conditions [6, 7], not many studies have evaluated the effect on patient-important outcomes under programmatic conditions outside the African region [8–12]. The national TB control programme of Indonesia adopted Xpert in March 2012 as a routine test for presumptive MDR-TB patients as part of their efforts to scale up services for programmatic management of drug-resistant TB (PMDT) [13]. The present study aimed to evaluate the effects of Xpert introduction upon TB and RIF resistance detection rates, treatment initiation rates and health system delays in three provincial public hospitals in Indonesia. A patient cohort tested with conventional diagnostics during one year pre-intervention (Year 1) was compared to a cohort tested with Xpert during one year post-intervention (Year 2). As a secondary objective, Xpert results were compared with culture and DST within the same individuals in Year 2, where the first diagnostic was used as initial test for TB and RR-TB and the latter as diagnostic workup to confirm MDR-TB.

## Study Population and Methods

Included were all individuals at risk of MDR-TB registered between 1 March 2011 and 31 March 2013 at three clinics offering PMDT services in West-, Central- and East-Java. Table 1 shows definitions of the nine mutually exclusive risk groups for MDR-TB according to Indonesian PMDT guidelines. In March 2012, no data were collected because sites transitioned from conventional to Xpert testing. The intervention involved four system changes: introduction of a new diagnostic algorithm; introduction of revised test request form, laboratory and clinical registers; installation of the Xpert machine, computer and uninterrupted power supply system; and training of laboratory and clinical staff. No changes were made with respect to human resources, funding for supplies and drugs, sample and result referral systems, or other processes. The conventional diagnostic approach was to collect one sputum sample from each individual and conduct smear microscopy and culture on solid or liquid media. If culture was positive for TB, an isolate was re-cultured for first-line DST. After the intervention, one sputum sample was collected for Xpert testing and a second sample was used for diagnostic workup with culture and first-line DST. Two out of three sites sent samples for culture and DST to a reference laboratory using liquid media, while the third site was linked to a laboratory using solid media. Guidelines dictated that second-line MDR-TB treatment was to start immediately after an Xpert RR-TB result was obtained and if necessary, adjusted later according to DST results.

**Table 1:** Definitions of nine groups at risk of multidrug-resistant TB in line with Indonesian guidelines for programmatic management of drug-resistant TB.

Risk category	Definition
1 Chronic cases, mostly patients who failed first-line TB re-treatment	Patients who are still sputum smear-positive at the end of first-line TB re-treatment (Category 2)
2 Patients on first-line TB re-treatment without smear conversion	Patients on first-line TB re-treatment (Category 2) who test smear-positive after three months of treatment
3 Patients with previous TB treatment outside of national program	Patients who received any type of TB treatment outside of the national programme, e.g. non-DOTS or private clinics
4 Patients who failed first-line TB treatment	Patients who are still sputum smear-positive at the end of first-line TB treatment (Category-1)
5 Patients on first-line TB treatment without smear conversion	Patients on first-line TB treatment (Category 1) who test smear-positive after three months of treatment
6 Relapse cases	Patients whose most recent treatment outcome (Category 1 or 2) was ‘cured’ or ‘treatment completed’ and return with symptoms of TB
7 Patients returning after loss to follow-up	Patients who interrupted any type of TB treatment for two or more consecutive months and return with symptoms of TB
8 Close contacts of MDR-TB patients	People living in the same household or spending many hours a day in the same indoor living space of an MDR-TB patient and who show symptoms of TB
9 Patients co-infected with HIV and TB	Patients who tested positive for HIV and TB with diagnostic tests

*Abbreviations*: *TB*, *tuberculosis; DOTS*, *direct observed treatment strategy; MDR-TB*, *multidrug-resistant tuberculosis; HIV*, *human immunodeficiency virus*.

Primary data sources were the electronic presumptive client registers. Individual patient data were transferred to a database in Microsoft Excel: client registration date, identification number, sex, birth date (or age), address, referring health facility, MDR-TB risk group, specimen identification number, smear microscopy/culture/DST/Xpert result and release date, and treatment registration number and start date. Laboratory registers were used to verify and complete test results and dates. Treatment registers and consultations with PMDT doctors helped to validate and complete treatment information. The various registers were linked by matching unique identification numbers or sex, age and address. Inconsistencies were discussed with laboratory and hospital staff and solved on-site. After registration, individuals were followed for at least three months to record treatment start, culture and DST results. Records were excluded if individuals were entered double (first record was discarded as it usually contained a failed test result), or if second-line treatment was started before testing. Data analysis was done using IBM SPSS Statistics 21. Differences in TB and RR-TB positivity rates and second-line treatment initiation rates between Year 1 and 2 were compared using the Pearson Chi square test with a 5% significance level. Median time between registration, test result release and treatment start before and after the intervention were compared using the Mood’s Median test with a 95% confidence interval (CI) and 5% significance level. Censored cases were RR-TB cases not treated at the end of the observation period. Comparing Xpert results with culture and DST as a diagnostic workup in Year 2 was done by calculating the percentage of agreement (concordance) of Xpert and culture to detect TB, and of Xpert and DST to detect RIF resistance.

Ethical clearance for this study was received from the Health Research Ethics Committee at the Medical Faculty of Padjadjaran University in Bandung, West-Java, Indonesia under number 181/UN6.C2.1.2/KEPK/PN/2012. Informed consent was not obtained as the intervention was done as a programmatic change in routine care. Further, data were de-identified and analyzed anonymously.

## Results

**Fig 1:**
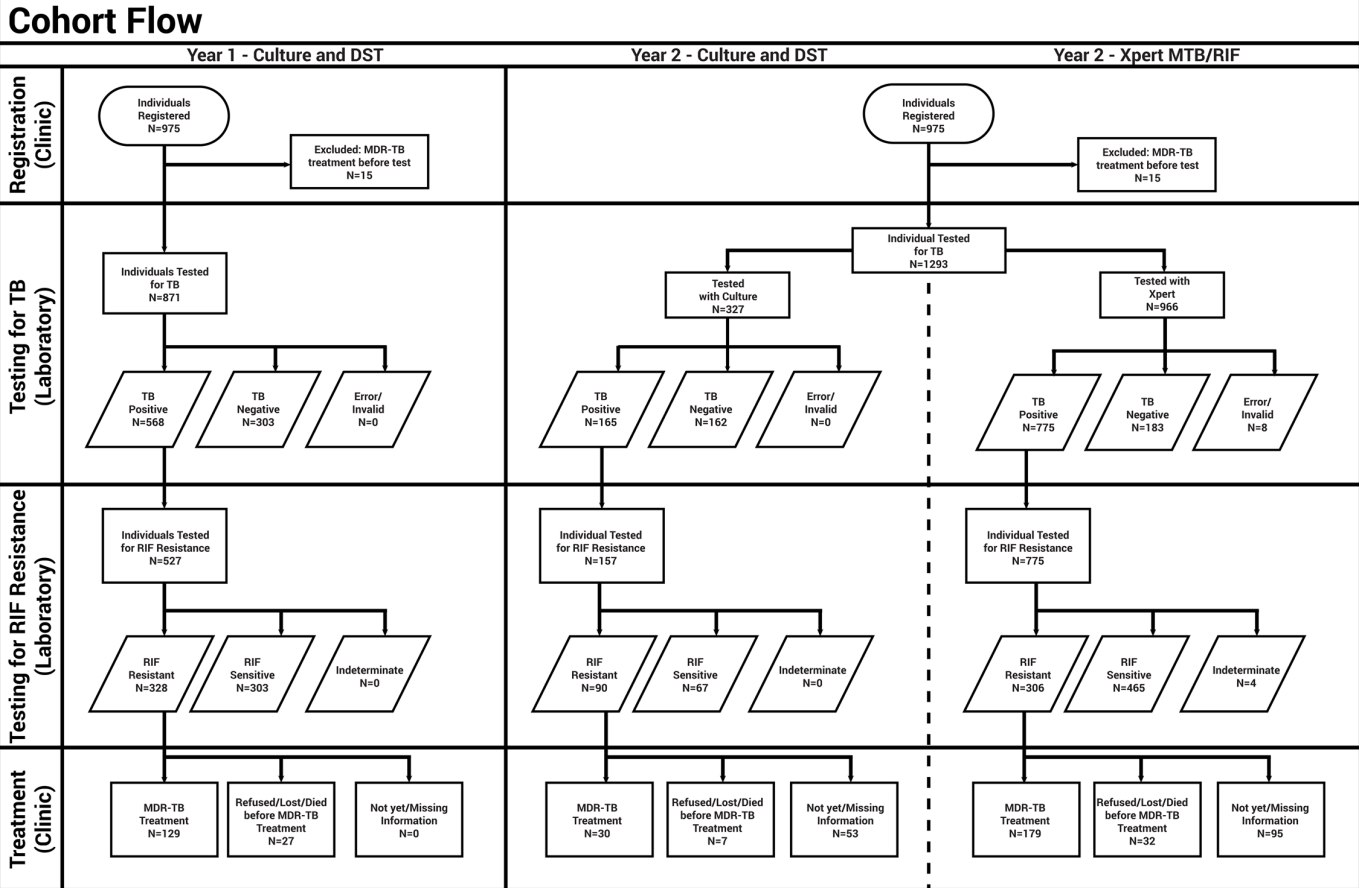
Diagnosis and treatment of individuals at risk of multidrug-resistant pulmonary TB at three provincial hospitals in Java, Indonesia. The flowchart shows the number of tested, detected and treated individuals in two cohort years. From March 2011 to Feb 2012 (Year 1), individuals were tested with conventional culture and drug-susceptibility testing. From March 2012 to February 2013 (Year 2), individuals were tested with culture and drug-susceptibility testing or Xpert MTB/RIF. *Abbreviations*: *TB*, *tuberculosis; DST*, *drug-susceptibility testing; RIF*, *rifampicin; MDR-TB*, *multidrug-resistant TB; N*, *number*.

After excluding double entries (n = 6 before, n = 2 after), a total of 975 individuals were registered in the year before (Year 1) and 1,442 in the year after (Year 2) introduction of Xpert, which is a relative increase of 47.9% (Fig 1). In Year 2 only 998 (69.2%) individuals were tested with Xpert, because of a three-month stock-out of tests (cartridges) from mid-August to mid-October 2012. During that period 327 people (22.7%) were tested with culture and DST instead and analyzed separately. Population characteristics were similar in Year 1 and 2 in terms of gender and age and differed slightly with regard to MDR-TB risk group (Table 2). However, both years saw most individuals being categorized as relapse cases after completing Category-1 or -2TB treatment. The only significant difference was the location of referring health care facilities: 6% more individuals were sent from within the same facility where testing was done (e.g. TB ward or out-patient department), while 8% fewer people were referred from other facilities in the same district.

**Table 2:** Characteristics of individuals at risk of multidrug-resistant pulmonary TB registered from March 2011 to February 2012 (Year 1) and March 2012 to February 2013 (Year 2) at three provincial hospitals in Java, Indonesia.

	Pre-intervention (Year 1, all cases)	Post-intervention (Year 2, all cases)	Prop. diff. (Chi square)^a^	Post-intervention (Year 2, cases tested with Xpert)	Prop. diff. (Chi square)^b^
	n (%)	n (%)	p-value	n (%)	p-value
**Total registered individuals at risk of MDR-TB**	975 (100)	1442 (100)		998 (100)	
Sex			0.921		0.663
Male	589 (60.4)	872 (60.5)		610 (61.1)	
Female	386 (39.6)	555 (38.5)		375 (37.6)	
Missing	0 (0)	15 (1.0)		13 (1.3)	
Age group			0.744		0.690
0–15	6 (0.6)	7 (0.5)		5 (0.5)	
16–30	207 (21.2)	323 (22.4)		225 (22.5)	
31–45	351 (36.0)	535 (37.1)		348 (34.9)	
46–60	303 (31.1)	442 (30.7)		325 (32.6)	
>60	106 (10.9)	109 (7.5)		76 (7.6)	
Missing	2 (0.2)	26 (1.8)		19 (1.9)	
MDR-TB criteria			0.036		0.013
1 Chronic cases, mostly patients who failed first-line TB re-treatment	173 (17.7)	217 (15.0)		159 (16.0)	
2 Patients on first-line TB re-treatment without smear conversion	45 (4.6)	63 (4.4)		40 (4.0)	
3 Patients reporting previous TB treatment outside of national program	41 (4.2)	59 (4.1)		41 (4.1)	
4 Patients who failed first-line TB treatment	149 (15.3)	203 (14.1)		140 (14.0)	
5 Patients on first-line TB treatment without smear conversion	83 (8.5)	165 (11.4)		117 (11.7)	
6 Relapse cases	342 (35.1)	494 (34.3)		340 (34.1)	
7 Patients returning after loss to follow-up	122 (12.5)	189 (13.1)		124 (12.4)	
8 Close contacts of MDR-TB patients	10 (1.0)	10 (0.7)		6 (0.6)	
9 Patients co-infected with HIV and TB	8 (0.8)	35 (2.4)		29 (2.9)	
Missing	2 (0.2)	7 (0.5)		2 (0.2)	
Referral facility			<0.001		<0.001
Came by themselves	186 (19.1)	247 (17.1)		180 (18.0)	
Same hospital	93 (9.5)	227 (15.7)		158 (15.8)	
Hospital in same district	393 (40.3)	497 (34.5)		323 (32.4)	
Hospital in other district	302 (31.0)	469 (32.5)		336 (33.7)	
Missing	1 (0.1)	2 (0.1)		1 (0.1)	

^a^ Proportional difference between all cases in the pre- and post-intervention year.

^b^ Proportional difference between all cases in the pre-intervention year and cases tested with Xpert in the post-intervention year.

*Abbreviations*: *Xpert*, *Xpert MTB/RIF assay; TB*, *tuberculosis; MDR-TB*, *multidrug-resistant tuberculosis; HIV*, *human immunodeficiency virus*.

In further analysis, 15 (1.5%) and 32 (2.2%) people were excluded from Year 1 and 2 respectively, because they received second-line treatment before being tested. In Year 1 and 2, 90.7% (871/960) and 91.7% (1,293/1,410) of individuals received a diagnostic test for TB, respectively. Test and treatment results for individuals tested with and without Xpert in Year 2 were compared to Year 1 as different cohorts (S1 Table). When individuals tested with culture in Year 1 and 2 were compared, the proportion that tested TB positive decreased by 14.7% from 65.2% to 50.5% (p<0.001), but no other significant differences were observed. When individuals tested with culture in Year 1 were compared with those tested with Xpert in Year 2, differences were more pronounced. First, TB positivity rate increased by 15.0% from 65.2% to 80.2% (p<0.001). Secondly, RIF resistance rate decreased by 18.2% from 57.3% to 39.5% (p<0.001) among TB positive patients and by 6.8% from 38.5% to 31.7% (p<0.001) among all tested. The total number of RR-TB cases was similar. Thirdly, the proportion of RR-TB patients that started second-line treatment increased by 19.2% from 39.3% to 58.5% (p<0.001) and the proportion without information on treatment initiation declined by 19.4% from 52.4% to 31.0% (p<0.001). Medical staff confirmed that most patients without treatment information were contacted multiple times without response and had likely not started second-line treatment. No significant difference was found in the proportion of RR-TB patients that deceased, refused, returned to their local clinic, were lost before treatment start or not eligible for second-line treatment. Out of the 101 Xpert RR-TB patients that did not start treatment, 55 (54.5%) had a follow-on culture result. Among them, 43 (78.2%) were culture positive for TB; and of those 38 (88.4%) were DST RIF resistant.

**Fig 2:**
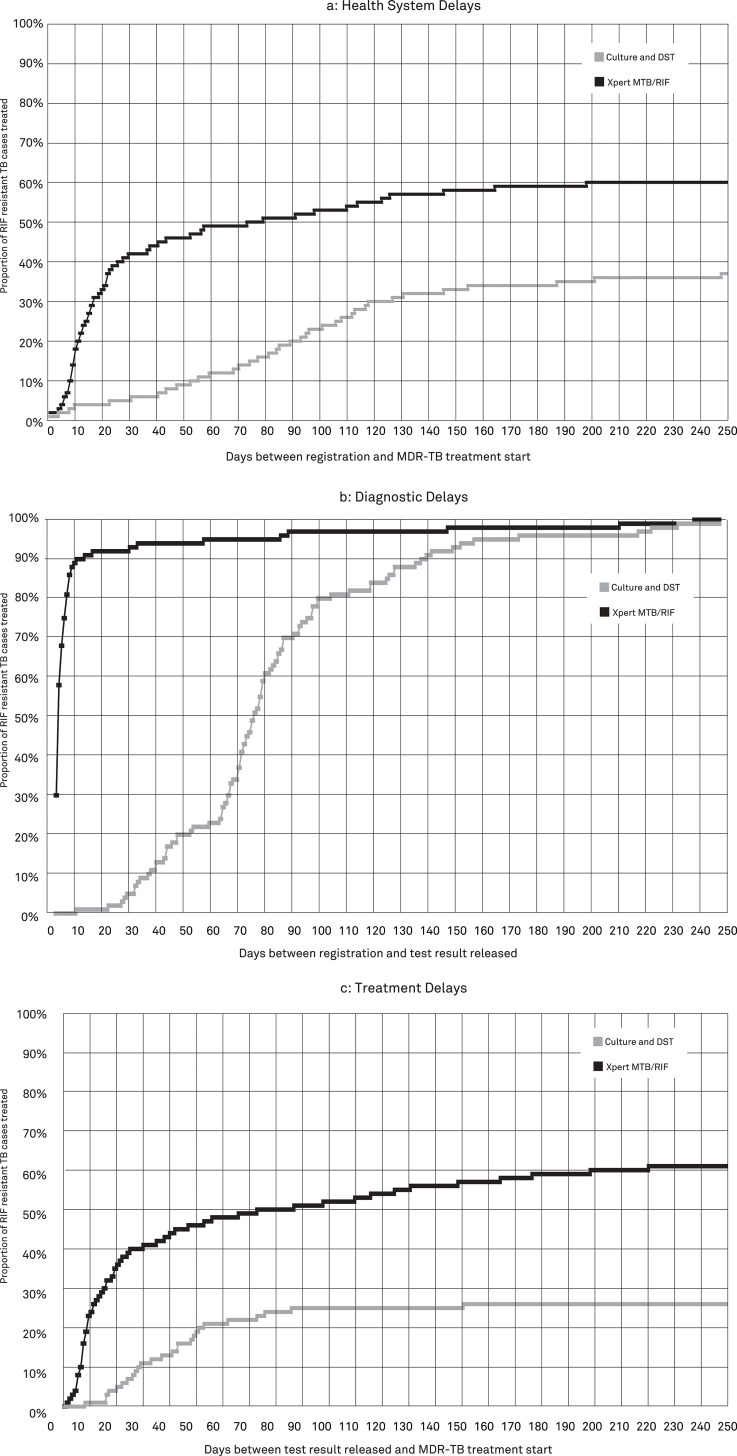
Time to diagnosis and treatment of rifampicin-resistant TB patients in three provincial hospitals in Java, Indonesia. These figures show Kaplan Meier time-to-event graphs of health system delays (a), diagnostic delays (b) and treatment delays (c) for rifampicin resistance TB patients detected with culture drug-susceptibility testing from March 2011 to February 2012 and Xpert MTB/RIF from March 2012 to February 2013. *Abbreviations*: *TB*, *tuberculosis; DST*, *drug-susceptibility testing; RIF*, *rifampicin*.

Delays in detection and treatment of RR-TB were compared among those tested with culture and DST in Year 1 versus those tested by Xpert in Year 2. One month after initial registration of a person at risk of MDR-TB, 6% of patients diagnosed with conventional DST and 42% of those diagnosed with Xpert were started on second-line treatment (Fig 2A). Expressed as median time delays, the time from presumptive client registration to treatment initiation was reduced by 72.0 days (p<0.001), from a median of 88.0 days (IQR 51.0–116.0) when culture and DST was used to a median of 16.0 days (IQR 9.0–41.0) when Xpert was used. The decrease in time from client registration to treatment initiation was mostly attributable to the decrease in time from registration to test result release, which was reduced by a median of 74.0 days (p<0.001) from 75.0 days (IQR 63.0–95.0) to 1.0 day (IQR 0.0–4.0) (Fig 2B). Similarly, the time to start of second-line treatment after release of a RR-TB diagnostic result was reduced by a median of 27.0 days (p = 0.005) from 42.0 days (IQR 25.0–55.0) to 15.0 days (IQR 7.0–51.0) (Fig 2C).

In Year 2, 70.8% (549/775) of Xpert TB positives and 57.4% (105/183) of Xpert TB negatives received culture and DST workup (Table 3). Concordance between the two diagnostic approaches to detect TB was 65.0% (425/654) and to detect RIF resistance 89.3% (300/336). Notably, 38.2% (210/549) of Xpert TB positives tested culture negative and among them 61.0% (128/210) had a negative smear microscopy result. This rate was highest among patients that had no smear conversion after three months of Category 1 or 2 TB treatment or who failed Category 1 treatment; in other words, patients that were recently treated (Table 4). Further, 18.1% (19/105) of Xpert TB negatives tested culture positive and among them 31.6% (6/19) had a positive smear microscopy result and 63.2% (12/19) were DST RIF resistant.

**Table 3:** Culture and drug-susceptibility testing results following Xpert MTB/RIF testing within the same individuals at risk of multidrug-resistant TB in three provincial hospitals in Java, Indonesia.

	Culture and DST						
	TB positive RIF resistant	TB positive RIF sensitive	TB positive RIF indeterminate	TB negative (including NTM)	Sub-total	Missing culture result	Total
Xpert MTB/RIF	n (%)	n (%)	n (%)	n (%)	n (%)	n	n
TB positive RIF resistant	158 (71.5)	18 (8.1)	0 (0)	45 (20.4)	221 (100)	85	306
TB positive RIF sensitive	18 (5.5)	142 (43.4)	3 (3.9)	164 (50.2)	327 (100)	138	465
TB positive RIF indeterminate	0 (0)	0 (0)	0 (0)	1 (100)	1 (100)	3	4
TB negative	12 (11.4)	4 (3.8)	3 (2.9)	86 (81.9)	105 (100)	78	183
Error	0 (0)	0 (0)	0 (0)	2 (100)	2 (100)	6	8
Total	188 (28.7)	164 (25.0)	6 (0.9)	298 (45.4)	656 (100)	310	966

*Abbreviations*: *DST*, *drug-susceptibility testing; TB*, *tuberculosis; RIF*, *rifampicin; NTM*, *non-tuberculosis mycobacteria*.

**Table 4:** Follow-on culture results among individuals from various risk groups that tested Xpert MTB/RIF TB positive in three provincial hospitals in Java, Indonesia.

	**Culture Result**				
	Negative	TB positive	NTM	Contaminated	Total
Xpert TB positive individuals (by risk criteria)	n (%)	n (%)	n (%)	n (%)	n (%)
Chronic cases, mostly patients who failed first-line TB re-treatment	27 (29.7)	63 (69.2)	0 (0)	1 (1.1)	91 (100)
Patients on first-line TB re-treatment without smear conversion	14 (51.9)	11 (40.7)	1 (3.7)	1 (3.7)	27 (100)
Patients with previous TB treatment outside of the national program	4 (16.0)	21 (84.0)	0 (0)	0 (0)	25 (100)
Patients who failed first-line TB treatment	33 (48.5)	34 (50.0)	1 (1.5)	0 (0.0)	68 (100)
Patients on first-line TB treatment without smear conversion	57 (70.4)	20 (24.7)	4 (4.9)	0 (0)	81 (100)
Relapse cases	50 (25.9)	140 (72.5)	2 (1.0)	1 (0.5)	193 (100)
Patients returning after loss to follow-up	12 (20.3)	43 (72.9)	2 (3.4)	2 (3.4)	59 (100)
Close contacts of MDR-TB patients	1 (25.0)	3 (75.0)	0 (0)	0 (0)	4 (100)
Patients co-infected with HIV and TB	2 (40.0)	3 (60.0)	0 (0)	0 (0)	5 (100)
**Total**	**200 (36.2)**	**338 (61.1)**	**10 (1.8)**	**5 (0.9)**	**553 (100)**

*Abbreviations*: *Xpert*, *Xpert MTB/RIF assay; TB*, *tuberculosis; NTM*, *non-tuberculosis mycobacteria; HIV*, *human immunodeficiency virus*.

## Discussion

Xpert was introduced in Indonesia with the main aim to shorten time to diagnosis and treatment of MDR-TB patients. This study found that post-intervention time to treatment was greatly reduced by almost 2.5 months, likely as a result of a shorter time for testing with Xpert than with culture and DST. Also, it took less time to send Xpert results back to clinicians, because the test was done in the PMDT hospital laboratory itself while culture and DST was performed in a reference laboratory located some distance away from the clinic. Another important finding was that after Xpert was introduced, a considerably larger proportion of RR-TB patients started second-line MDR-TB treatment. It is probable that this was an effect of the more rapidly available result of Xpert. Nevertheless, the treatment initiation rate remained below 60%. Some of the missing patients could have returned to local clinics and remained on first-line treatment, while others could have been switched from second- to first-line treatment on the basis of follow-on culture and DST results. These patients should have been classified as lost or referred, but this was often not recorded. This reflects an urgent gap between diagnosis and treatment and the need to strengthen patient registration, follow-up and monitoring alongside the introduction of Xpert. This finding adds onto recent concerns about patient management after Xpert diagnosis expressed in global guidance [3].

Concordance between Xpert and culture as a diagnostic workup post-intervention was limited due to a substantial proportion of Xpert TB positive culture TB negative test results. This may represent the occurrence of false-negative culture results caused by unviable bacilli in sputum samples as a result of over-decontamination or delays in transportation or inoculation. Laboratory staff confirmed that delays in culture inoculation after receiving samples were a problem throughout Year 2 due to shortage of staff in a reference laboratory serving two of the three Xpert sites. This could also explain why the culture TB positivity rate decreased by 14.7% from Year 1 to Year 2. Alternatively, some tests could have been false-positive for TB with Xpert as a result of the different nature of genotypic versus phenotypic tests and the fact that Most individuals had recent previous TB treatment. It is likely that after multiple first-line treatment cycles, retreatment patients had either dead or damaged bacteria in their lungs that are positively identified on a molecular test like Xpert, but cannot grow on culture. This would be in line with recent findings from South Africa [14] and could also explain why the TB positive rate of Xpert in Year 2 was higher compared to that of culture in Year 2 but the RIF resistance rate was not.

Overall agreement between Xpert and phenotypic DST for detection of RIF resistance was good. In some cases, Xpert could have been false-negative for RIF as it has a sensitivity for RIF resistance of 95% [4]. Xpert false-positive RIF resistance results could also have occurred as was reported by previous studies [15, 16]. However, recent work showed that over 10% of RIF resistance mutations detected by molecular methods like Xpert may not be detected by phenotypic DST, especially not with liquid culture, while they do result in low-level resistance and worse outcomes on first-line TB treatment for previously treated patients [17, 18]. This means that the one-tenth of Xpert RIF resistant patients that were DST RIF sensitive could have been truly RIF resistant. DNA sequencing techniques would help clarify discordance between both diagnostic methods in the future.

This study was unique in that it evaluated the effects of Xpert within a strictly programmatic setting: besides the physical placement of the new test, revision of registers and training of medical staff, national TB guidelines were unchanged. However, a programmatic intervention study involves inherent limitations.

The main limitation was that the pre-post intervention design might have introduced selection bias and performance bias. Immediately and consistently after Xpert was introduced almost 50% more individuals at risk of MDR-TB were tested than the year before. Clinicians confirmed that the excitement of a new rapid test led to more attention for PMDT and increased referral of presumptive MDR-TB patients, which may have influenced the type of individuals being sent for testing as well as the treatment decision-making process. Although no significant shift in MDR-TB risk groups was observed post-intervention, it is still possible that clinicians sent presumptive MDR-TB clients earlier for diagnosis, e.g. presumptive relapse patients at the first suspicion of TB symptoms instead of at a second or third patient visit. This would explain why the total number of RR-TB diagnosed cases remained similar in Year 1 and 2. Further, it cannot be excluded that the increase in treatment initiation rate and reduction in time delays were partly caused by clinicians tracing patients more actively to start treatment, in addition to the rapid turn-around-time of Xpert results.

A second limitation was the large proportion of missing follow-on culture and DST results among Xpert-tested individuals in Year 2, which could have introduced partial verification bias. If culture was considered as the gold standard and we would correct for bias using a population TB prevalence of 54.6%, a possible overestimation of concordance to detect TB of 4.6% (60.4% vs. 65.0%) was found. In particular, the proportion of Xpert TB negative culture TB positive patients may have been underestimated by as much as 15.8% (33.9% vs. 18.1%). Missing culture results were due to delays in culture inoculation as mentioned above and results becoming available only beyond the period of data collection.

## Conclusions

The results of this study indicate that the introduction of Xpert has helped to start more RR-TB patients on second-line treatment and initiate treatment sooner as compared to using conventional culture and DST. Fast turn-around of results likely resulted in less drop-out during the diagnostic process. In addition, clinician’s treatment decision-making could have been positively influenced by the excitement of a new rapid test. Still, the overall proportion of RR-TB patients that started second-line treatment was low and merits special attention from the national TB control program. Proper engagement of clients at high risk of MDR-TB is essential to prevent pre-treatment loss to follow-up. Further analysis is needed to clarify the increase in TB case detection and decrease in RIF resistance using Xpert compared to culture and DST. In conclusion, Xpert holds promise to improve RR-TB case finding and treatment in Indonesia, but its implementation should coincide with improved patient management to optimize PMDT services.

## Supporting Information

S1 Table Diagnosis and treatment of individuals at risk of multidrug-resistant pulmonary TB tested with culture and drug-susceptibility testing in Year 1, and culture and drug-susceptibility testing or Xpert MTB/RIF in Year 2 at three provincial hospitals in Java, Indonesia. (DOCX)
