# Supplementary material for: Effects of Introducing Xpert MTB/RIF on Diagnosis and Treatment of Drug-Resistant Tuberculosis Patients in Indonesia: A Pre-Post Intervention Study
Source: PLoS One. 2015 Jun 15;10(6):e0123536. doi: 10.1371/journal.pone.0123536 (PMC4468115; doi:10.1371/journal.pone.0123536)
Supplement: S1 Table — (DOCX) [file pone.0123536.s001.docx]

|  | Pre-intervention (Year 1, all cases) | | Post-intervention (Year 2, cases tested with culture and DST) | | Prop. diff.  (Chi square) | Post-intervention (Year 2, cases tested with Xpert) | | Prop. diff.  (Chi square) |
| --- | --- | --- | --- | --- | --- | --- | --- | --- |
|  | n | % | n | % | p-value | n | % | p-value |
| Tested for TB | 871 | 100 | 327 | 100 |  | 966 | 100 |  |
| TB positive | 568 | 65.2 | 165 | 50.5 | <0.001 | 775 | 80.2 | <0.001 |
| TB negative (incl. NTM) | 303 | 34.7 | 162 | 49.5 | <0.001 | 183 | 18.9 | <0.001 |
| Error or invalid | 0^a^ | 0 | 0 | 0 | 1 | 8 | 0.8 | n/a |
| TB positives tested for resistance | 527 |  | 157 |  |  | 775 |  |  |
| Rifampicin resistant | 328 | 62.2 | 90 | 57.3 | 0.30 | 306 | 39.5 | <0.001 |
| Rifampicin sensitive | 199 | 37.8 | 67 | 42.7 | 0.30 | 465 | 60.0 | <0.001 |
| Indeterminate | 0^b^ | 0 | 0 | 0^b^ | n/a | 4 | 0.5 | n/a |
| Rifampicin resistant cases tested for isoniazid resistance | 326 |  | 90 |  |  | 176 |  |  |
| Isoniazid resistant | 304 | 93.3 | 86 | 95.6 | 0.50 | 153  (9 RIF sen.) | 86.9 | 0.05 |
| Isoniazid sensitive | 22 | 6.7 | 4 | 4.4 | 0.50 | 23  (9 RIF sen.) | 13.1 | 0.05 |
| Rifampicin resistant cases | 328 |  | 90 |  |  | 306 |  |  |
| Died before treatment | 8 | 2.4 | 2 | 2.2 | 0.95 | 3 | 1.0 | 0.50 |
| Lost before treatment | 3 | 0.9 | 2 | 2.2 | 0.50 | 7 | 2.3 | 0.30 |
| Refused treatment | 6 | 1.8 | 2 | 2.2 | 0.90 | 10 | 3.3 | 0.70 |
| Denied treatment | 1 | 0.3 | 0 | 0 | 0.70 | 0 | 0 | 0.50 |
| Not MDR-TB, referred back | 9 | 2.7 | 1 | 1.1 | 0.50 | 6 | 2.0 | 0.70 |
| Considered not to have TB (culture neg.) | 0 | 0 | 0 | 0 | n/a | 6 | 2.0 | n/a |
| Started 2^nd^ line treatment | 129 | 39.3 | 30 | 33.3 | 0.30 | 179 | 58.5 | <0.001 |
| No(t) yet/Missing | 172 | 52.4 | 53 | 58.9 | 0.30 | 95 | 31.0 | <0.001 |

^a^ It is likely that some individuals without a culture result in fact had a contaminated culture, but this was not recorded in laboratory registers.

^b^ It is likely that some culture positive isolates had a contaminated DST tests, but this was not recorded in laboratory registers.

*Abbreviations: TB, tuberculosis; NTM, non-tuberculosis mycobacteria; RIF, rifampicin; MDR-TB, multidrug-resistant tuberculosis.*
